# Functional roles of ornithine decarboxylase and arginine decarboxylase during the peri-implantation period of pregnancy in sheep

**DOI:** 10.1186/s40104-017-0225-x

**Published:** 2018-01-24

**Authors:** Yasser Y. Lenis, Gregory A. Johnson, Xiaoqiu Wang, Wendy W. Tang, Kathrin A. Dunlap, M. Carey Satterfield, Guoyao Wu, Thomas R. Hansen, Fuller W. Bazer

**Affiliations:** 1 Department of Animal Science, Texas A&M University, College Station, TX 77843-2471 USA; 2 Center for Animal Biotechnology and Genomics, Texas A&M University, College Station, TX 77843 USA; 3 Centauro Research Group, School of Veterinary Medicine, Faculty of Agrarian Science, Universidad de Antioquia, Calle 70 No, 52-21 Medellín, Colombia; 4 Faculty of Agricultural Sciences, Calle 222 No. 55-37, UDCA, Bogota, Colombia; 5 Present address: National Institute of Environmental Health Sciences, Research Triangle Park, NC 27709 USA; 6 Animal Reproduction and Biotechnology Laboratory, Department of Biomedical Sciences, College of Veterinary Medicine and Biomedical Sciences, Colorado State University, Fort Collins, CO 80523 USA

**Keywords:** Agmatine, Arginine, Interferon tau, Polyamines, Trophectoderm cells

## Abstract

**Background:**

Polyamines stimulate DNA transcription and mRNA translation for protein synthesis in trophectoderm cells, as well as proliferation and migration of cells; therefore, they are essential for development and survival of conceptuses (embryo/fetus and placenta). The ovine conceptus produces polyamines via classical and non-classical pathways. In the classical pathway, arginine (Arg) is transformed into ornithine, which is then decarboxylated by ornithine decarboxylase (ODC1) to produce putrescine which is the substrate for the production of spermidine and spermine. In the non-classical pathway, Arg is converted to agmatine (Agm) by arginine decarboxylase (ADC), and Agm is converted to putrescine by agmatinase (AGMAT).

**Methods:**

Morpholino antisense oligonucleotides (MAOs) were designed and synthesized to inhibit translational initiation of the mRNAs for ODC1 and ADC, in ovine conceptuses.

**Results:**

The morphologies of MAO control, MAO-ODC1, and MAO-ADC conceptuses were normal. Double knockdown of *ODC1* and *ADC* (MAO-ODC1:ADC) resulted in two phenotypes of conceptuses; 33% of conceptuses appeared to be morphologically and functionally normal (phenotype a) and 67% of the conceptuses presented an abnormal morphology and functionality (phenotype b). Furthermore, MAO-ODC1:ADC (a) conceptuses had greater tissue concentrations of Agm, putrescine, and spermidine than MAO control conceptuses, while MAO-ODC1:ADC (b) conceptuses only had greater tissue concentrations of Agm . Uterine flushes from ewes with MAO-ODC1:ADC (a) had greater amounts of arginine, aspartate, tyrosine, citrulline, lysine, phenylalanine, isoleucine, leucine, and glutamine, while uterine flushes of ewes with MAO-ODC1:ADC (b) conceptuses had lower amount of putrescine, spermidine, spermine, alanine, aspartate, glutamine, tyrosine, phenylalanine, isoleucine, leucine, and lysine.

**Conclusions:**

The double-knockdown of translation of *ODC1* and *ADC* mRNAs was most detrimental to conceptus development and their production of interferon tau (IFNT). Agm, polyamines, amino acids, and adequate secretion of IFNT are critical for establishment and maintenance of pregnancy during the peri-implantation period of gestation in sheep.

**Electronic supplementary material:**

The online version of this article (10.1186/s40104-017-0225-x) contains supplementary material, which is available to authorized users.

## Background

During the peri-implantation period of pregnancy, ovine conceptuses undergo dramatic morphological changes from spherical to tubular and filamentous forms which requires histotroph. Histotroph is a general term used to describe fluid in the uterine lumen that contains molecules synthesized and secreted by uterine lumenal (LE), superficial glandular (sGE), and glandular (GE) epithelia, as well as hormones, enzymes, cytokines, glucose, adhesion proteins, growth factors, polyamines and amino acids that are selectively transported into the uterine lumen [1, 2]. Histotroph not only guarantees nutritional support for conceptus development, but it also has an important role in enhancing production of interferon tau (IFNT) by trophectoderm cells. Conceptuses in the uterine gland knockout ewes do not transition from spherical to tubular and filamentous forms between Days 11 and 14 of pregnancy, and secretion of IFNT is insufficient for the establishment of pregnancy [3]. IFNT is the antiluteolytic pregnancy recognition signal in ruminants for maintenance of a functional corpus luteum required for production of progesterone. IFNT and progesterone act in concert to induce and stimulate the expression of genes in the ovine uterus such as those for transport of amino acids and glucose. Therefore, the secretion of IFNT is a measure of the functionality of the trophectoderm following knockdown of translation of mRNAs for ODC1 and ADC individually and in combination. When IFNT secretion is compromised, the transport of nutrients into the uterine lumen is also compromised. Histotrophic nutrition during pregnancy results from the collective effects of progesterone and IFNT, as well as placental lactogen, and placental growth hormone [4].Polyamines (putrescine, spermidine and spermine) are small, aliphatic, polycationic biogenic molecules that have carbon chains of varying length and different numbers of amino groups. Polyamines are very important molecules with multiple roles that include: 1) stabilization of DNA and DNA transcription; 2) stabilization of mRNA and mRNA translation for protein synthesis; 3) growth, proliferation and migration of cells; 4) stability of cell membranes; 5) binding ATP; 6) ion channel functions; and 7) receptor-ligand interactions [5–7]. Therefore, polyamines are essential for growth, development and survival of mammalian conceptuses [8]. Arginine (Arg), a conditionally essential amino acid for conceptus survival, growth and development [9], is a precursor for important biological molecules including urea, creatinine, ornithine, proline, nitric oxide, agmatine (Agm) and polyamines [10]. Arg is a very versatile amino acid, representing 14% of total nitrogen in body proteins. It stimulates the secretion of growth hormone and insulin, and the expression of important genes such as insulin like growth factor 2, nitric oxide, and ornithine decarboxylase. Furthermore, Arg activates mechanistic targeting of rapamycin (mTOR) cell signaling pathways to induce proliferation, migration and adhesion of ovine trophectoderm cells required for implantation, survival and growth of blastocysts, as well as survival, growth, and health of mammalian conceptuses [11–16].

Agmatine, a product of the decarboxylation of Arg, was discovered in 1994 in bovine and rat brains. Agmatine is considered a neurotransmitter or neuromodulator with neuroprotective and cardioprotective effects and it is a precursor for synthesis of polyamines [17–20]. We reported that Agm increases expression of mRNAs for SLC7A1 (Arg transporter), AGMAT and 5-azacytidine induced protein 2 (AZI2) in ovine trophectoderm cells (oTr1) cells [21]. Thus, Agm is an important molecule for production of polyamines in conceptuses, but it may also have additional functions within the pregnant uterus of sheep [21, 22].

In vivo experiments revealed that during the pre-implantation period of pregnancy (d 10 to 16), the amounts of Arg, glutamine, and leucine in the uterine lumen increased 7-, 6- and 5-fold, respectively compared with non-pregnant sheep [5]. In addition, the amounts of ornithine and Arg in ovine allantoic fluid increased significantly between d 30 and 60 of gestation and that suggested their importance as precursors for the synthesis of polyamines during the establishment and maintenance of pregnancy, as well as for normal development of the conceptus during pregnancy [5].

The ovine conceptus produces polyamines via two different pathways: the classical and the non-classical pathway [21, 23]. In the classical pathway, Arg is transformed into ornithine, which is then decarboxylated by ornithine decarboxylase (ODC1) to produce putrescine, which is the substrate for spermidine synthase to generate spermidine and spermidine is catabolized to spermine by spermine synthase. In the non-classical pathway, Arg is converted to Agm by ADC and Agm is converted to putrescine by AGMAT. In the peri-implantation period of pregnancy the main pathway to produce polyamines is considered to be the classical pathway; however, previous reports showed that the non-classical pathway was activated in vivo in ovine conceptuses when translation of *ODC1* mRNA was inhibited using a morpholino antisense oligonucleotide [23]. Interestingly, the compensatory non-classical pathway was activated in only one-half of the ovine conceptuses deficient in *ODC1* mRNA translation. Only those conceptuses that activated the ADC/AGMAT pathway developed normally. It was concluded that the conversion of Arg into ornithine and then putrescine via the arginase-ODC1 pathway was the primary pathway for synthesis of polyamines and that the ADC/AGMAT pathway was a secondary pathway for producing polyamines by ovine conceptuses. Therefore, the present experiments examined the hypothesis that effects of in vivo knockdown of translation of both *ODC1* and *ADC* and their combination in ovine conceptuses would be embryonic lethal due to the inability of the conceptus to synthesize polyamines from ornithine by knockdown of translation of *ODC1* mRNA or the inability of the conceptus to produce agmatine from arginine due to knockdown of translation of *ADC* mRNA.

## Methods

### Animal model

Estrous cycles were synchronized in multiparous Rambouillet ewes (*n* = 30) using a commercially available Eazi-Breed controlled intra-uterine drug release (CIDR;Pfizer, New York) device for 12 d followed by intramuscular injection of 20 mg Lutalyse (Pfizer, New York) at the time of CIDR removal. Estrus (d 0) was detected by a vasectomized ram and ewes were subsequently mated to intact rams of known fertility. All experimental and surgical procedures were approved by the Institutional Animal Care and Use Committee at Texas A&M University.

### Morpholino design

Morpholino antisense oligonucleotides (MAOs) were designed and synthesized to inhibit translational initiation of the mRNAs for ODC1 and ADC, which are enzymes involved in the synthesis of polyamines via the classical and non-classical pathways. The *MAO-ODC1* had the sequence 5’-ACTCTTCATTACCAAAGTTGTTCAT-3′ and targeted the starting codon of *ODC1* mRNA (GenBank accession no. NM_002539). For *ADC,* the sequence was 5’ TTTCTCTCAGGTAGCCAGCCATGCC’3 (GenBank accession no. NM_001293722.1) and targeted the starting codon of *ADC*. The *MAO* control (Gene Tools) had the sequence 5’-CCTCTTACCTCAGTTACAATTTATA-3′ and targeted a splice site mutant of *Homo sapiens* hemoglobin *β-chain* (HBB) gene (GenBank accession no. GU324922). All morpholinos were synthesized with a 3′-lissamine (fluorescent tag) to facilitate their detection in trophectoderm [23].

### Experimental design and tissue collection

For morpholino delivery into the uterine lumen, we performed a medial laparotomy on d 8 post-mating and delivered the morpholino into the lumen of the uterine horn ipsilateral to the corpus luteum (CL) as previously described [23]. Briefly, a small incision was made in the oviduct just above the tubouterine junction and a small catheter was passed through the incision and into the uterine lumen. The catheter was attached to a 1-mL syringe containing the morpholino and the morpholino was delivered from the syringe into the uterine lumen. The contralateral uterine horn was ligated near the uterine body to prevent migration of the conceptus into that uterine horn. The ewes were assigned randomly to the following treatments: MAO control (*n* = 7); MAO-ODC1 (*n* = 7); MAO-ADC (*n* = 8); or MAO-ODC1:MAO-ADC (*n* = 8). Development and implantation of ovine conceptuses was not affected by surgery or morpholino delivery [23, 24]. MAO control, MAO-ODC1, and MAO-ADC were complexed to lissamine as described previously [23]. The MAO combination (100 nmol MAO-ODC1 plus 100 nmol MAO-ADC) was prepared with Gene Tools Endo-Porter delivery reagent (200 μL) and diluted to 1.2 mL final volume with OPTI-MEM (Invitrogen; Grand Island, NY, U.S.A.). All MAOs were injected once into the lumen of the uterine horn (cranial portion) ipsilateral to the ovary with a corpus luteum (CL). On d 16, ewes were ovariohysterectomized to obtain conceptuses, uterine flushings and uterine and conceptus tissues. The ligated uterine horn ipsilateral to the CL was flushed with 10 mL sterile phosphate-buffered saline (PBS), pH 7.2. The presence or absence of a functional CL and the presence of a conceptus in the uterine flushing was recorded and pregnancy rate determined for each treatment group. For pregnant ewes with a conceptus, the morphology of the conceptus was recorded as small, thin, fragile, fragmented, elongated, and/or normal. After photographing each conceptus using a digital camera, the conceptus was removed from the uterine flush with a transfer pipette, and the recovered volume of uterine flushing recorded. Portions of each conceptus and sections (0.5 cm) from the mid-portion of the uterine horn ipsilateral to the CL were placed in optimal-cutting temperature compound (Fisher Health Care, Houston, Texas), frozen in liquid nitrogen and stored at −80 °C or fixed in freshly prepared 4% (wt/vol) paraformaldehyde in PBS, pH 7.2, for 48 h and then in 70% ethanol for 24 h. The uterine flushing was centrifuged (5,000 × *g* for 15 min), aliquoted, and stored at −80 °C until analyzed.

### Slot blot assay of IFNT

We performed slot blot analyses to quantify the abundance of IFNT in uterine flushings as described previously [21]. For this assay, 10 μg of protein from each uterine flush was used to optimize analyses by slot blotting. The signal for each sample was expressed relative to the immunoreactivity of proteins in uterine flushings from MAO control ewes. The data are expressed as IFNT intensity in uterine flushes per 10 μg total protein. The abundance for IFNT was quantified by measuring the intensity of light emitted from correctly sized bands under ultraviolet light using a ChemiDoc EQ system and Quantity One software (Bio-Rad, Hercules, CA).

### Radioimmunoassay analyses of IFNT

A radioimmunoassay (RIA) was used to measure concentration of IFNT in uterine flushings [23]. This assay was developed and validated at Colorado State University [25]. The intra- and inter-assay coefficients of variation were 11.3% and 14.2%, respectively.

### Quantitative Immunofluorescence microscopy

Lissamine-labeled MAOs were analyzed by fluorescence microscopy to confirm their effective delivery into the trophectoderm. Cryosections of the OCT containing conceptuses (10 μm) as well as uteri (10 μm) were prepared and placed directly into mounting medium containing 4′, 6-diamidino-2-phenylindole (DAPI) to visualize the nuclei. Also, the translational knockdown efficiency of MAO-ODC1 and MAO-ADC and their combination was evaluated in frozen sections of conceptuses or uteri by immunofluorescence microscopy as previously described [23, 26, 27]. For each primary antibody, images were captured with identical microscope and detector settings to facilitate comparisons of spatial distribution and fluorescence intensities among samples from ewes on the various treatments. Signals were quantified using Image J software (Version 1.47, National Institutes of Health) and standardized procedures described previously [28].

### Analyses for Putrescine, Spermidine, Spermine, Agmatine, and amino acids

Concentrations of polyamines, as well as agmatine and amino acids were determined in uterine flushings and conceptuses using procedures described previously [29]. Briefly uterine flushings (100 μL), and conceptuses (15 mg) were acidified with 100 μL of 1.5 mol/L HClO_4_ and neutralized with 50 μL of 2 mol/L K_2_CO_3_. The neutralized extracts were analyzed for polyamines, amino acids, and agmatine after making 1:2.5 dilutions for conceptuses and no dilution for uterine flushes. Amino acids and polyamines were subjected to high-performance liquid chromatography analyses involving precolumn derivatization with o-phthaldialdehyde (OPA) reagent I or II. Reagent I preparation was used for polyamines and agmatine and reagent II was used for amino acid assays and prepared as described previously [23].

### Glucose assay

Concentrations of glucose in uterine flushings were determined enzymatically using a fluorometric method involving hexokinase and glucose-6-phosphate dehydrogenase [30, 31]. All samples were acidified and neutralized with 1.5 mol/L HClO_4_ and 2 mol/L K_2_CO_3_, respectively. The total dilution factor was 2.5. The absorbances A1 and A2 were measured at 340 nm to obtain the final absorbance (A2-A1), and the final absorbance was extrapolated according to the standard curve. The data are expressed as total glucose (volume of uterine flush × concentration of glucose/mL; nmol) in uterine flushes.

### Statistical analyses

Results are presented as mean ± SEM. The statistical analyses were performed using one-way or two-way ANOVA and orthogonal contrast with the JPM software of the SAS Institute INC). Differences at *P* ≤ 0.05 were considered significant. Normality of data and homogeneity of variance were tested using the Shapiro-Wilk test and Brown-Forsythe test, respectively, in the Statistical Analysis System. The effect of treatment on pregnancy rates was analyzed using Chi-Square analysis.

## Results

### MAO delivery and effect of knockdown of translation of *ODC1* and *ADC* mRNAs in ovine conceptuses

**Fig. 1:**
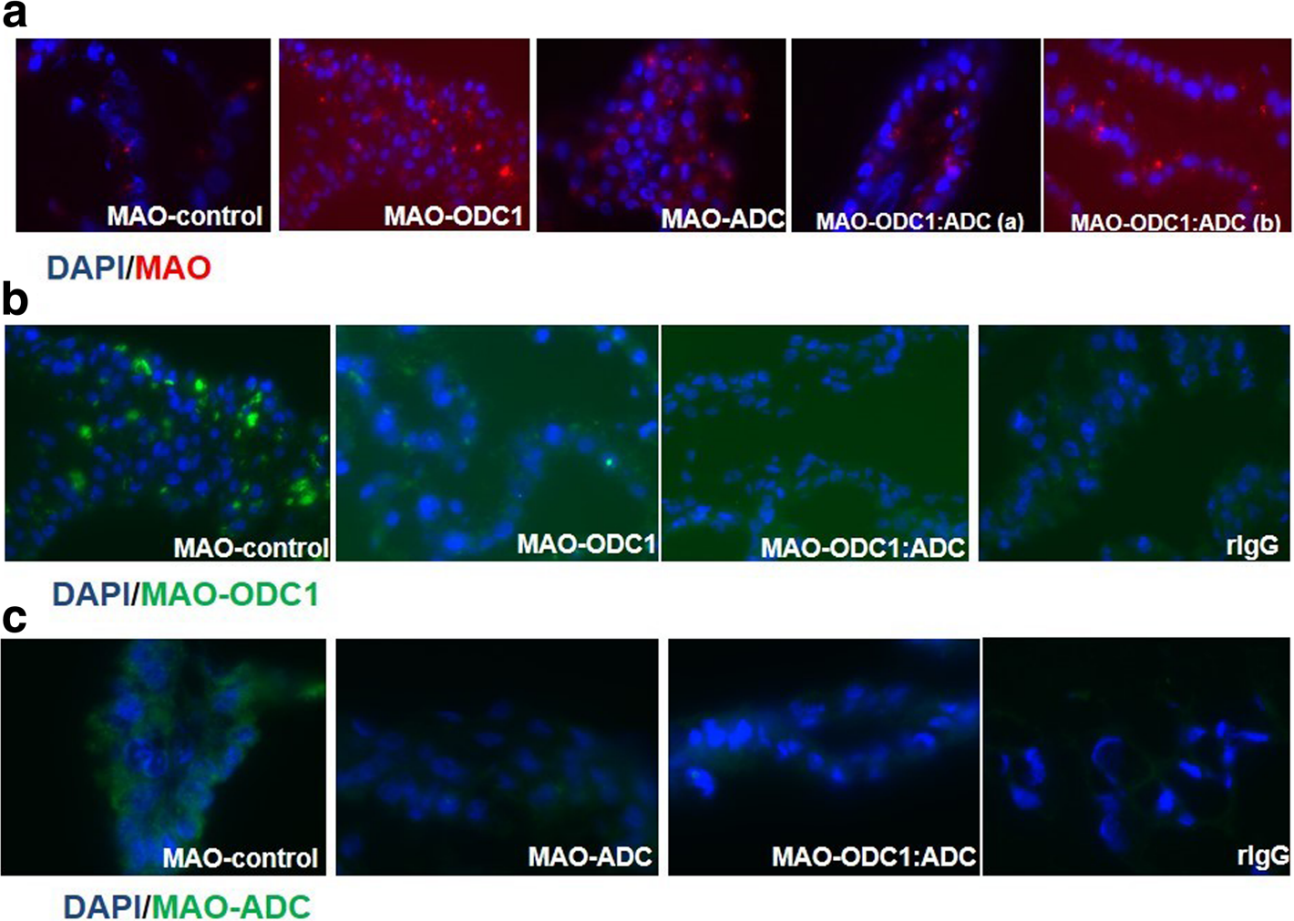
Evidence for delivery of MAOs and knockdown of translation of mRNAs for ODC1, ADC and their combination in ovine conceptus trophectoderm on d 16 of pregnancy. [**a**] These images confirmed uptake of lissamine-tagged MAOs (red staining) in MAO control, MAO-ODC1, MAO-ADC, MAO-ODC1: MAO-ADC (a) and MAO-ODC1: MAO-ADC (b). [**b**] The results indicate that translation of *ODC1* mRNA was not inhibited in MAO-control conceptuses (Panel B1) as noted by abundant green staining; however, green staining for ODC1 is very weak for the conceptuses exposed to MAO-ODC1, and MAO-ODC1:MAO-ADC. [**c**] Immunofluorescence images indicate abundant green staining for ADC in the MAO-contro conceptuses, but not in the MAO-ADC or MAO-ODC1:MAO-ADC conceptuses. These results confirmed that the respective MAOs inhibited translation of ODC1 and ADC as expected. Purified non-relevant rabbit IgG was substituted for the primary antibody as the negative control as evidenced by the lack of green staining

We confirmed MAO delivery and knockdown of translation of *ODC1* and *ADC* mRNAs independently and in combination using immunofluorescence microscopy. For each treatment group, MAO uptake by conceptuses was confirmed by the presence of 3′-lissamine tag in conceptus trophectoderm (Fig. 1A), but not uterine epithelia. Additionally, knockdown of detectable amounts of ODC1 and ADC proteins was confirmed by immunofluorescence analyses when compared with the abundance of those proteins in MAO control conceptuses (Fig. 1B, C). These results indicate that MAOs were delivered efficiently into trophectoderm cells of the conceptus and that knockdown for ODC1 and ADC proteins in trophectoderm cells was achieved.

### In vivo knockdown of translation of *ODC1* and *ADC* mRNAs individually and in combination affected the abundance of polyamines in the uterine lumen

**Fig. 2:**
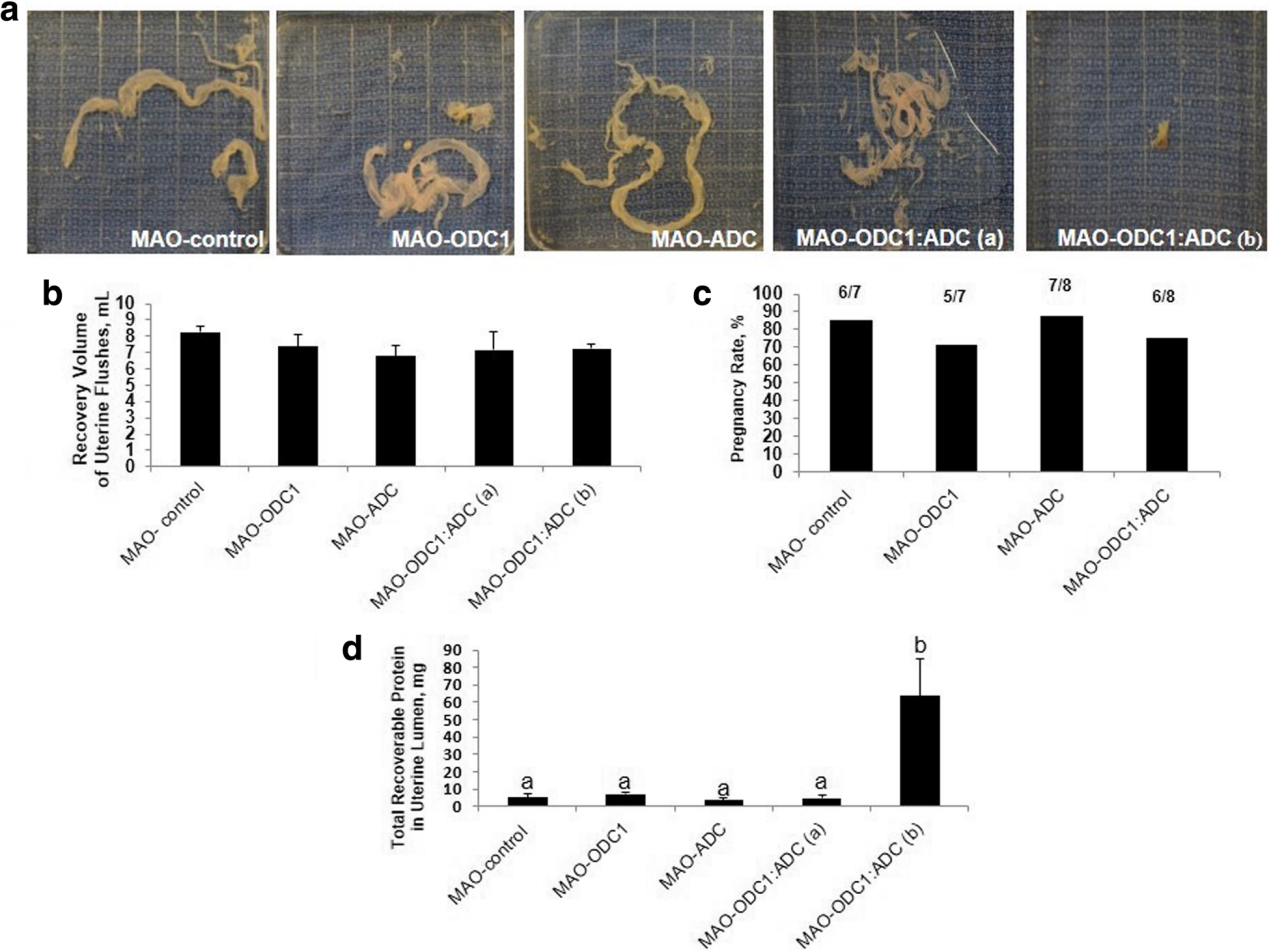
Gross morphology of ovine conceptuses on d 16 of pregnancy following knockdown of translation of mRNAs for ODC1, ADC and their combination (ODC1:ADC). [**a**] Compared to MAO-control conceptuses (Panel A1; *n* = 6), in vivo knockdown of translation of mRNAs for ODC1*-MAO* (Panel A2, *n* = 5) and ADC*-MAO* (Panel A3, *n* = 7) did not adversely affect development of the conceptuses. However, the combination knockdown of translation of both ODC1 and ADC (MAO-ODC1: MAO-ADC) resulted in two phenotype based on their morphological and functional development. The MAO-ODC1:MAO-ADC (a) conceptus phenotype (Panel A4, *n* = 2) was normal, healthy and elongated, while the MAO-ODC1:MAO-ADC (b) conceptus phenotype (Panel A5, *n* = 4) was abnormal, fragmented and not elongated. [**a**] Recovery volume of uterine flushes [**b**] and pregnancy rate [**c**] were not different (*P* > 0.05) among treatment groups. However total recoverable protein in uterine flushes from MAO-ODC1: MAO-ADC (b) was greater (*P* < 0.05) from values for the other treatment groups [**d**]. Means with different superscript letters were different (*P* < 0.05). Data are presented as means and SEM

Agmatine is an intermediate in the alternative pathway for synthesis of polyamines in ovine conceptus tissue. To evaluate the different biological effects of knockdown of *ODC1*, *ADC* and their combination on polyamine production by conceptuses, we measured the abundances of agmatine, spermidine, putrescine and spermine in uterine flushes. Total recoverable Agm was less abundant (*P* < 0.05) in uterine flushes from MAO-ODC1, MAO-ADC and MAO-ODC1:MAO-ADC ewes (a and b) compared with MAO control ewes (Fig. 2A). Total putrescine was less (*P* < 0.05) in uterine flushes from MAO-ADC and MAO-ODC1:MAO-ADC (b) than for MAO control ewes, but values were not different among MAO control, MAO-ODC1 and MAO-ODC1:ADC (a) ewes. The MAO-ODC1:ADC (b) ewes had less spermidine (*P* < 0.05) in uterine flushes. Spermine was less abundant (*P* < 0.05) in uterine flushes from MAO-ODC1, MAO-ADC and MAO-ODC1:MAO-ADC (a and b) compared with MAO control ewes (Fig. 2 B, C, D).

### In vivo knockdown of translation of mRNAs for ODC1 or ADC in trophectoderm of ovine conceptuses resulted in morphologically normal conceptuses; however, the double knockdown of translation of mRNAs for ODC1 and ADC resulted in morphologically abnormal phenotypes of conceptuses

**Fig. 3:**
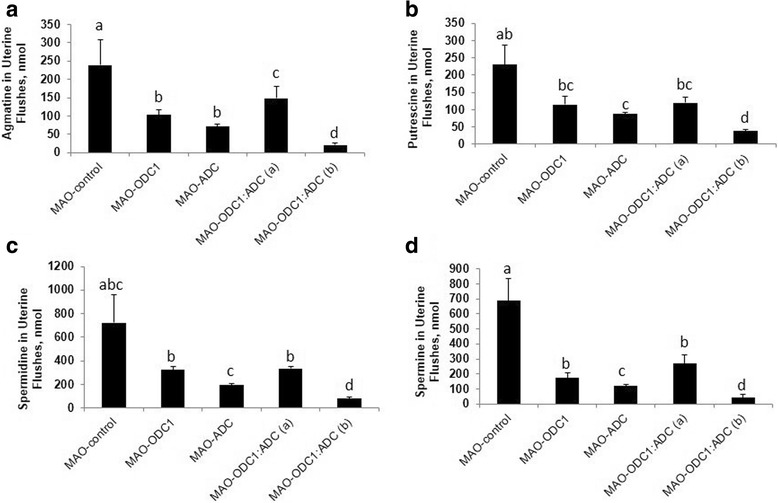
The abundances of agmatine, putrescine, spermidine and spermine in uterine flushings of ewes in which translation of mRNAs for ODC1, ADC and their combination (ODC1: ADC) was inhibited. In vivo knockdown of translation of *ODC1* mRNA (*n* = 5), *ADC* mRNA (*n* = 7), ODC1 and ADC (a) (*n* = 2) and ODC1 and ADC (b) (*n* = 4) decreased (*P* < 0.05) abundance of agmatine (nmol) [**a**] and spermine (nmol) [**d**] in uterine flushes. In vivo knockdown of translation or mRNA for both MAO and ADC resulted in phenotype MAO-ODC1:MAO-ADC (b) in which abundances of putrescine [**b**] and spermidine [**c**] were reduced in uterine flushes. Means with different superscript letters were different (*P* < 0.05). Data are presented as mean and SEM

We investigated in vivo translational knockdown of *ODC1*, *ADC* and their combination in conceptuses between d 8 and d 16 of pregnancy. The morphology of ovine conceptuses change from spherical on d 12, to tubular on d 13 and then filamentous on d 14 to d 16 of gestation. The morphologies of MAO control, MAO-ODC1 and MAO-ADC conceptuses were normal. However, the MAO-ODC1:MAO-ADC combination knockdown of mRNA translation resulted in two phenotypes of conceptuses. The first phenotype represented 33% (*n* = 2) of conceptuses that appeared morphologically and functionally normal (elongated and healthy) was designated MAO-ODC1:ADC (a). The second phenotype represented 67% (*n* = 4) of conceptuses and presented as abnormal morphologically and functionally (not elongated and fragmented) and this phenotype was designed MAO-ODC1:MAO-ADC (b) (Fig. 3A). Only two MAO-ODC1:ADC (b) conceptuses that were collected could be analyzed, but the others were completely fragmented.

**Fig. 4:**
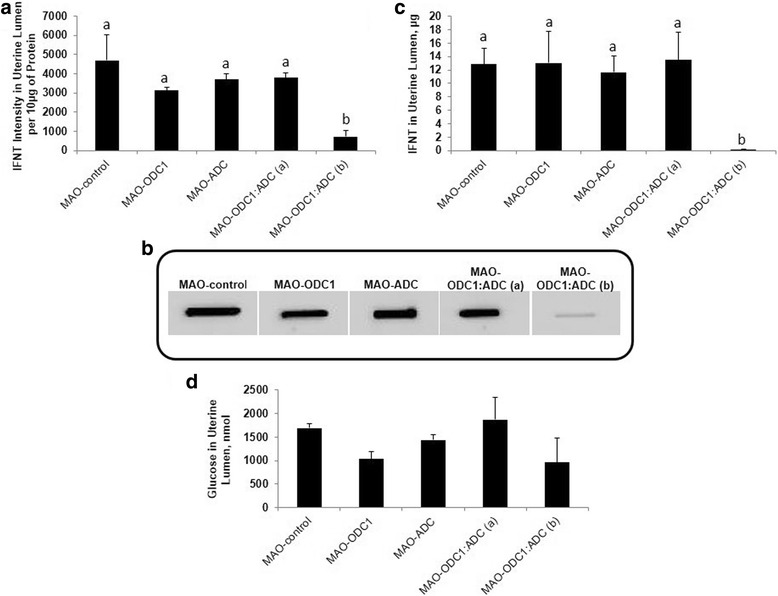
Total intensity and amount (μg) of interferon tau (IFNT) produced and secreted by ovine conceptus on d 16 of pregnancy and total amount (nmol) of glucose in uterine lumen. In vivo knockdown of ODC1 (*n* = 5), ADC (*n* = 7) and their combination phenotype (a) (*n* = 2) did not present significantly different (*P* > 0.05) in **a** and **b**), IFNT intensity per 10 μg of protein and in (**c**), total amount (μg) of IFNT compared with MAO control (*n* = 6). However the intensity and amount of IFNT from MAO-ODC1: ADC (b) (*n* = 4) was significantly (*P* < 0.05) less compared with MAO control. In vivo knockdown of ODC1, ADC, and ODC1:ADC (a) and (b) had not significant (*P* > 0.05) effects on (**d**), total glucose uterine flushes. Significant effects are indicate by different superscript letters (*P* < 0.05). Data are presented as mean and SEM

The pregnancy rates did not differ (*P* > 0.05) and was 80% overall compared to 86% for the MAO control, 72% for MAO-ODC1, 88% for MAO-ADC and 75% for the MAO-ODC1:MAO-ADC ewes. There was no significant effect of treatment on volume of uterine flushing recovered (Fig. 3B,C). Total protein was greater (*P* < 0.05) in uterine flushings from MAO-ODC1:ADC (b) ewes compared with MAO control, MAO-ODC1, MAO-ADC and MAO-ODC1:ADC (a) ewes (Fig. 3D). The relative abundance of IFNT in 10 μg of protein in uterine flushings was not different (*P* > 0.05) between MAO-ODC1, MAO-ADC and MAO-ODC1:MAO-ADC (a) compared with MAO control ewes. However, ewes with MAO-ODC1:ADC (b) conceptuses had less (*P* < 0.05) detectable IFNT in their uterine flushings. Those results were confirmed by IFNT RIA analysis, which did not detect differences in total IFNT in uterine flushes among MAO control, MAO-ODC1, MAO-ADC and MAO-ODC1:MAO-ADC (a) ewes, but total IFNT was less (*P* < 0.05) in uterine flushes from MAO-ODC1:ADC (b) ewes (Fig. 4A, B, C).

### In vivo knockdown of mRNA translation for ODC1, ADC and their combination did not affect total glucose in uterine flushes

The morphological changes in the ovine conceptus during the peri-implantation period requires adequate histotroph that includes amino acids, growth factors, hormones, cytokines, enzymes, polyamines, adhesion proteins and glucose as an energy source. There was no effect of treatment (*P* > 0.05) on total recoverable glucose in uterine flushings (Fig. 4D).

### In vivo knockdown of translation of *ODC1* and *ADC* mRNAs individually and in combination reduced the abundance of polyamines, agmatine and spermine, in conceptus tissue

**Fig. 5:**
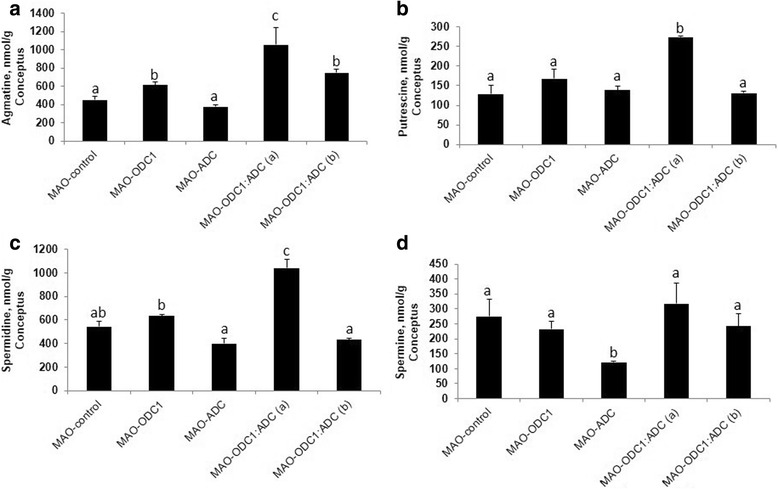
The abundances of agmatine, putrescine, spermidine and spermine in ovine conceptuses in which there was knockdown of translation of mRNAs for ODC1, ADC and their combination (ODC1: ADC). In vivo knockdown of ODC1 (*n* = 5), MAO-ODC1:ADC (a) (*n* = 2) and MAO-ODC1:MAO-ADC (b) (*n* = 4) increased agmatine (nmol/g) [**a**] in conceptus tissue. Knockdown of mRNAs for both ODC1 and ADC resulted in the MAO-ODC1: MAO-ADC (a) phenotype and tissues from those conceptuses had greater concentrations (nmol/g) of spermidine [**b**] and putrescine [**c**], but less spermine [**d**]. Means with different superscript letters are different (*P* < 0.05). Data are presented as mean and SEM

Agmatine was more abundant (*P* < 0.05) in conceptus tissue from MAO-ODC1, MAO-ODC1:MAO-ADC (a) and MAO-ODC1:ADC (b) compared with MAO control ewes (Fig. 5A). The amounts of spermidine and putrescine were greater (*P* < 0.05) in conceptus tissues from MAO-ODC1:ADC (a) ewes, while the abundance of spermine was lower (*P* < 0.05) in conceptus tissues from MAO-ADC ewes compared with that for all other treatment groups of ewes (Fig. 5B,C).

### In vivo knockdown of translation of *ODC1* and *ADC* mRNAs individually and in combination affected the abundances of amino acids in uterine flushes

High-performance liquid chromatography was used to determine abundances of amino acids in uterine flushes. Uterine flushings from MAO-ODC1:ADC (a) ewes had greater (*P* < 0.05) abundances of aspartate, glutamate, citrulline, arginine, tyrosine, phenylalanine, isoleucine leucine and lysine than for MAO control ewes. On the other hand, uterine flushes from MAO-ODC1:MAO-ADC (b) ewes had lower (*P* < 0.05) amounts of alanine, aspartate, glutamate, tyrosine, phenylalanine, isoleucine, leucine and lysine, but greater amounts of citrulline than for MAO control ewes. Uterine flushings from MAO-ODC1ewes had lower (*P* < 0.05) amounts of citrulline and tryptophan, while leucine and lysine were more abundant (*P* < 0.05) in uterine flushes from MAO-ADC compared to MAO control ewes. For uterine flushes from MAO-ODC1:MAO-ADC (a) ewes, there was more (*P* < 0.05) in aspartate (2.9-fold), glutamate (2-fold), citrulline (3-fold), arginine (1.6-fold), tyrosine (1.7-fold), phenylalanine (2.2-fold), isoleucine (1.6-fold), leucine (1.7-fold), and lysine (1.1-fold) compared with MAO control ewes. For uterine flushes from MAO-ODC1:MAO-ADC (b) ewes, there was less (*P* < 0.05) aspartate (1.8-fold), glutamate (2-fold), tyrosine (1.9-fold), phenylalanine (3.4-fold), isoleucine (2.8-fold), leucine (2.2-fold), and lysine (5.2-fold) than for uterine flushes from MAO control ewes (Table 1).

**Table 1:** Amino acids in uterine flushes, nmol/mL (Means ± SEM)

Amino acid	MAO-control	MAO-ODC	MAO-ADC	MAO-ODC1:ADC (a)	MAO-ODC1:ADC (b)
Asp	2.82 ± 1.00^a^	3.1 ± 0.7^a^	5.3 ± 1.7^a^	8.3 ± 1.8^b^	1.5 ± 0.1^c^
Glu	24.39 ± 8.16^a^	26.2 ± 4.8^a^	36.9 ± 9.1^ab^	50.1 ± 8.3^b^	12.4 ± 2.7^c^
Asn	9.88 ± 3.51^a^	7.1 ± 0.3^a^	11.2 ± 2.0^a^	20.9 ± 1.5^b^	4.3 ± 3.5^a^
Ser	25.33 ± 8.89	19.8 ± 2.4	40.1 ± 6.7	45.4 ± 11.9	35.9 ± 2.8
Gln	59.66 ± 28.58	34.1 ± 4.5	45.7 ± 9.7	70.1 ± 1.6	38.3 ± 26.9
His	16.87 ± 6.23	14.2 ± 1.6	21.1 ± 4.4	29.5 ± 12.3	7.6 ± 6.5
Gly	99.09 ± 28.59	109.0 ± 21.7	151.1 ± 19.3	135.6 ± 8.4	80.2 ± 39.8
Thr	17.96 ± 5.69	10.6 ± 1.7	18.2 ± 3.3	34.3 ± 1.4	11.2 ± 10.0
Cit	3.00 ± 1.22^a^	1.3 ± 0.2^b^	2.0 ± 0.5^ab^	9.2 ± 2.4^c^	10.1 ± 8.5^d^
Arg	14.08 ± 4.71^a^	10.0 ± 1.1^ab^	15.9 ± 3.5^ab^	23.6 ± 0.6^b^	16.8 ± 13.5^ab^
b-Ala	4.12 ± 1.47	3.7 ± 1.5	6.9 ± 3.5	6.8 ± 1.4	5.1 ± 3.5
Tau	23.63 ± 6.95	24.4 ± 4.8	33.2 ± 9.5	34.6 ± 8.2	58.3 ± 44.0
Ala	25.40 ± 9.94^ab^	22.4 ± 1.7^a^	36.7 ± 10.0^ab^	41.8 ± 4.1^b^	11.9 ± 3.8^c^
Tyr	7.33 ± 1.18^a^	6.6 ± 1.2^a^	8.6 ± 1.7^ab^	13.0 ± 2.0^b^	3.7 ± 1.4^c^
Trp	1.71 ± 0.21^a^	0.7 ± 0.1^b^	1.1 ± 0.4^ad^	1.0 ± 0.1^c^	2.1 ± 1.5^ad^
Met	0.44 ± 0.22	0.6 ± 0.2	1.0 ± 0.5	1.7 ± 0.8	0.6 ± 0.2
Val	8.33 ± 2.85	6.7 ± 0.5	8.0 ± 2.9	14.7 ± 3.1	3.6 ± 1.9
Phe	6.81 ± 1.61^a^	5.3 ± 1.7^a^	9.0 ± 2.7^a^	15.1 ± 3.0^b^	2.0 ± 1.0^c^
Ile	3.78 ± 1.16^a^	2.5 ± 0.3^a^	4.4 ± 1.1^ab^	6.1 ± 0.6^b^	1.3 ± 0.4^c^
Leu	8.53 ± 2.35^a^	6.5 ± 0.7^a^	10.2 ± 2.5^b^	14.8 ± 2.9^b^	3.7 ± 1.5^c^
Orn	10.71 ± 3.35	6.6 ± 1.6	10.7 ± 1.7	16.9 ± 3.8	6.4 ± 4.0
Lys	57.91 ± 2.22^a^	36.5 ± 5.1^a^	47.3 ± 13.9^b^	64.6 ± 3.8^c^	11.0 ± 7.9^d^

Amino acids in uterine flushes (nmol/mL). MAO-Control *n* = 4, MAO-ODC1 *n* = 3, MAO-ADC *n* = 5, MAO-ODC1: ADC (a) *n* = 2, MAO-ODC1: ADC (b) *n* = 2. All quantitative data are presented as mean and SEM. Legend: ornithine decarboxylase (ODC1); arginine decarboxylase (ADC). Values with different superscript letters are different (*P*<0.05)

## Discussion

Embryonic death in mammals ranges from 20 to 40%, with two-thirds occurring during the peri-implantation period of pregnancy [1, 2]. IFNT produced between d 10 to d 21 of pregnancy is the maternal recognition of pregnancy signal in sheep which ensures maintenance of the corpus luteum on the ovary and its production of progesterone [32, 33]. During the peri-implantation period, IFNT acts in concert with P4 to increase the expression of genes in uterine lumenal and superficial glandular epithelia to produce histotroph [4, 34, 35]. Histotroph is critical for the morphological changes (spherical to tubular to filamentous forms) in ovine conceptus development required for successful implantation [23, 36–38] (Additional file 1: Figure S3). Implantation in sheep is initiated by filamentous conceptuses as they complete elongation and there is attachment and adhesion of trophectoderm to the uterine luminal epithelium [36, 38]. Successful implantation and conceptus development require histotroph [4, 36, 39].

Ornithine decarboxylase and ADC are the rate-controlling enzymes in the classical and non-classical pathways, respectively, for production of polyamines by the ovine conceptus [5, 23]. Putrescine, spermidine and spermine are critical regulators of proliferation, migration, and differentiation of trophectoderm cells during the peri-implantation period of pregnancy [5, 23, 40]. To our knowledge, this is the first report of in vivo knockdown of translation of mRNAs for ODC1, ADC, and their combination. In the present study, knockdown of translation of *ODC1* and *ADC* mRNAs individually in conceptus trophectoderm did not significantly disrupt development of the conceptus or its production of IFNT. Interestingly, MAO-ODC1 conceptuses had a greater amount of Agm suggesting that the ADC/AGMAT pathway was active and functional for production of polyamines. Accordingly, Agm is converted to putrescine, spermidine and spermine to rescue the phenotype of ovine conceptuses in which translation of *ODC1* mRNA is blocked. On the other hand, Agm in MAO-ADC conceptuses was not different from that in MAO-control conceptuses. This result confirm that the main pathway for production of polyamines in ovine conceptuses is via ODC1 (arginine-ornithine-putrescine) [23].

Importantly, we determined that double in vivo knockdown of translation of both *ODC1* and *ADC* mRNAs in conceptus trophectoderm disrupted development and production of IFNT. Only 33% of the MAO-ODC1:MAO-ADC(a) conceptuses elongated and exhibited somewhat normal morphological development, while 67% of the MAO-ODC1:MAO-ODC1 (b) conceptuses failed to elongate and they exhibited an abnormal morphological phenotype. Further, MAO-ODC1:ADC (a) conceptuses produced 34-fold more IFNT than MAO-ODC1:ADC (b) conceptuses (13.5 μg versus 0.4 μg, respectively). Thus, the amount of IFNT in the uterine lumen is an indicator of the integrity and functionality of the conceptus trophectoderm (Fig. 4A) [32]. An explanation for the increase in protein in uterine flushings containing MAO-ODC1:ADC (b) conceptuses is not apparent, but it may reflect the greater degree of fragmentation of conceptuses with that phenotype and, therefore, more soluble protein in uterine flushings.

Interestingly, MAO-ODC1:MAO-ADC (a) conceptuses had greater amounts of Agm, putrescine and spermidine than abnormal MAO-ODC1:MAO-ADC (b) conceptuses which could, in part, explain the differences in morphology between MAO-ODC1:MAO-ADC (a) and MAO-ODC1:ADC (b) conceptuses. There is evidence that AGMAT is more abundant in uterine LE between d 14 and 16 of pregnancy (A. Nonato, K. Dunlap, Y. Lenin, F.W Bazer, unpublished data), which may explain the presence of Agm in histotroph can be taken up by trophectoderm cells and used to produce polyamines. Our results indicate that amounts of Agm in histotroph are associated positively with growth and development of the ovine conceptus as it can be used to synthesize polyamines required for conceptus development.

We reported that Agm increases expression of AGMAT in a positive feed-back manner, as well as *AIZ2* mRNAs to increase the amount of putrescine in trophectoderm cells [21]. Mouse embryos have a growth advantage compared with control embryos when cultured in the presence of putrescine [41]. And, oral supplementation of putrescine in the peri-ovulatory period improves oocyte quality, increases the number of cells in blastocysts and reduces embryonic deaths in mice [42].

Knockdown of *SLC7A1* (arginine transporter) retards development of ovine conceptuses as arginine is used to synthesize ornithine via ODC1 [22]. Fifty percent of sheep conceptuses in which translation of *ODC1* mRNA was blocked failed to develop morphologically and functionally, whereas 50% of the conceptuses did elongate and produced IFNT normally. Conceptuses that were normal had increased transcription of genes for AGMAT and ADC, but those conceptuses that did survive had a significantly greater abundance of AGMAT protein. Results of the present study confirm that activation of the ADC/AGMAT pathway is critical for growth and development of MAO-ODC1 conceptuses based on their ability to elongate and produce IFNT [23].

The amounts of Agm, spermidine, spermine, and amino acids in uterine flushings were affected by treatment in the present study. The abundance of glucose in uterine flushes increase 6-fold between d 10 and 15 of ovine pregnancy [43]. However, in the present study, in-vivo knockdown of translation of mRNAs for ODC1, ADC and their combination in conceptus trophectoderm did not affect the abundance of glucose in the uterine lumen. Thus, the MAOs did not affect uterine epithelia that express glucose transporters in response to progesterone and IFNT as reported previously [43].

Polyamines and Agm are important molecules during the peri-implantation period of pregnancy in sheep. For MAO-ODC1, MAO-ODC1:ADC (a) and MAO-ODC1:ADC (b) the significant decrease in Agm in uterine flushings could be due to greater uptake of Agm by the trophectoderm of ovine conceptus tissue for production of polyamines (ADC/AGMAT). However, MAO-ODC1:MAO-ADC (b) ewes had less putrescine, spermidine, and spermine in uterine flushes which may explain the failure of those conceptuses to elongate and produce IFNT. Rumen bacteria may contribute to agmatine in blood and in uterine fluid. Zhao et al. [44] reported that expression of *ODC1* mRNA at implantation sites in rats increased in response to estradiol-17β (100 ng/mouse). The expression of ODC1 antizyme 1 and spermidine/spermine N1-acetyltransferase also increased in uterine epithelial cells at those implantation sites in response to polyamines [44]. Arg is an important component of histotroph and a precursor for synthesis of polyamines in the classical and non-classical pathways [23]. Supplemental Arg has significant physiological effects to enhance reproductive performance in women, rats and gilts [45–48].

The abundance of Arg increases 8- to 13-fold in uterine histotroph of ewes during the peri-implantation period of pregnancy [47, 48] and that it stimulates migration, proliferation, and gene expression by trophectoderm cells [49]. In the present study, uterine flushes from ewes with MAO-ODC1:MAO-ADC (a) conceptuses had 1.6-fold more Arg than uterine flushes from ewes with MAO control conceptuses. This is important because Arg and polyamines activate the mTORC1 pathway and Arg enhances secretion of IFNT by trophectoderm cells of ovine conceptuses [50]. Interferon tau also induces expression of cationic amino acid transporters to deliver more Arg into the uterine lumen which enhances development of the ovine conceptus [2]. In the present study, there was a significant decrease in alanine, aspartate, glutamate, tyrosine, phenylalanine, isoleucine, leucine and lysine in uterine flushings of ewes with MAO-ODC1:MAO-ADC(b) conceptuses which could account for failure of those conceptuses to secrete sufficient amounts of IFNT [27, 43].

The amounts of Arg, glutamine and leucine are 7-, 6- and 5-fold greater, respectively in uterine flushes from Day 15 of pregnancy compared with cyclic ewes, indicating the importance of those amino acids during the peri-implantation period of pregnancy [43]. Arginine, leucine and glycine, stimulate the mTOR signaling pathway to increase translation of mRNAs for IFNT, nitric oxide synthases 2 and 3 (NO_2_ and NO_3_) and guanosine triphosphate cyclohydrolase [27, 43, 51]. Those results indicate the importance of increases in Arg and leucine in uterine flushes of ewes with MAO-ODC1:MAO-ADC (a) conceptuses.

**Fig. 6:**
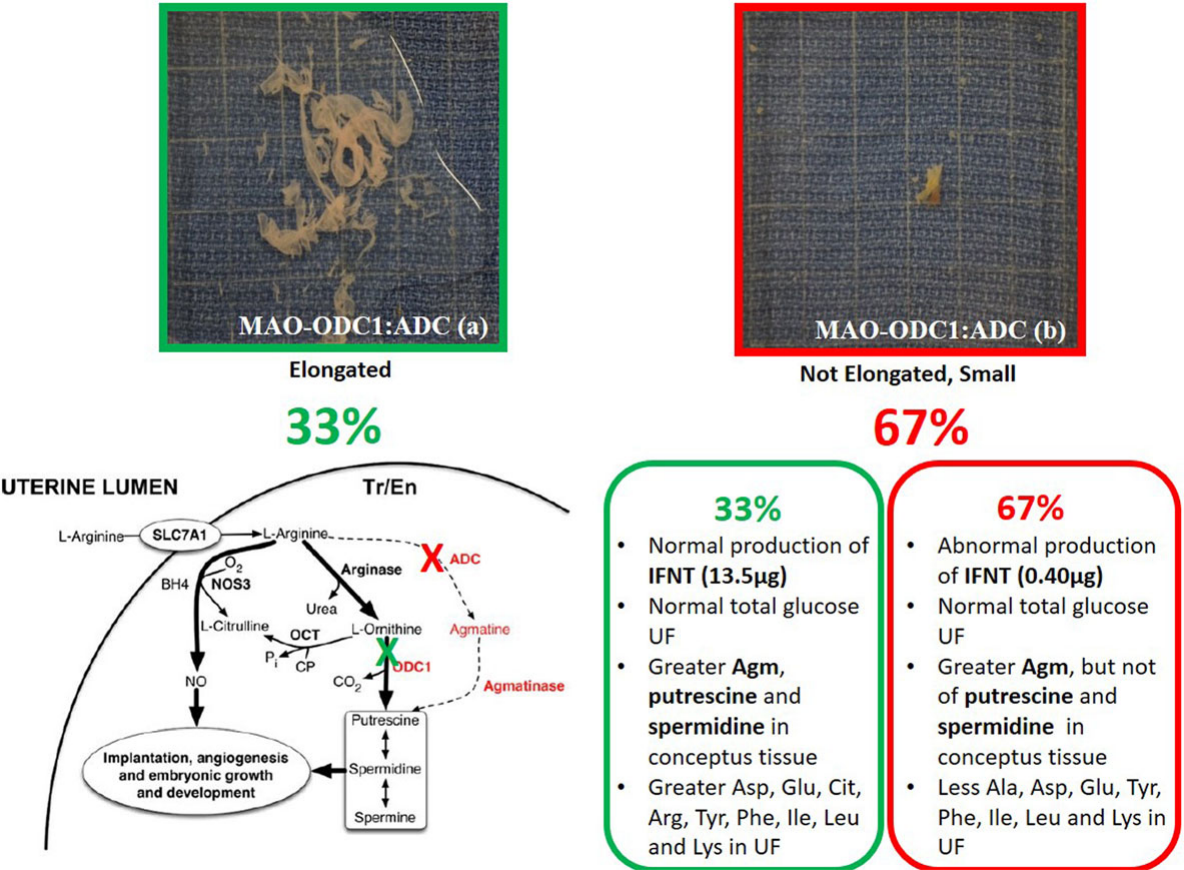
Knockdown of translation of both *ODC1* and *ADC* mRNAs resulted in two phenotypes of conceptuses: 33 % had the (MAO-ODC1:MAO-ADC (**a**) normal phenotype, while 66 % of MAO-ODC1:MAO-ADC (**b**) conceptuses had an abnormal phenotype with respect to morphology, production of interferon tau (IFNT), and and amino acids and polyamines in uterine flushings. MAO-ODC1:ADC (**a**) conceptuses had an elongated morphology with normal production of IFNT, greater amount of agmatine (Agm), putrescine, and spermidine in conceptus tissue, and greater amount of aspartate (Asp), glutamate (Glu), citrulline (Cit), arginine (Arg), tyrosine (Tyr), phenylalanine (Phe), isoleucine (Ile), leucine (Leu) and lysine (Lys) in uterine flushings than was the case for MAO-ODC1:MAO-ADC (**b**) conceptuses. The pathways involving ODC1 and ADC for synthesis of nitric oxide and polyamines is noted and their locations are noted by the green X and the red X, respectively

The cellular metabolism of polyamines is regulated by a family of antienzymes (AZI) that inactivate ODC1 and maintain homeostasis for polyamines within cells. The activity of ODC1 in vivo and in vitro can be stimulated by amino acids. However, the role of the amino acids in the uterine lumen, other than arginine, methionine and proline [52, 53] to serve as substrates for or influence synthesis of polyamines during the peri-implantation period of pregnancy in sheep is unclear (Fig. 6).

## Conclusions

Physiological levels of Agm, polyamines, amino acids in the uterine lumen, and adequate secretion of IFNT during the peri-implantation period of pregnancy are critical for a successful outcome of pregnancy in sheep. In vivo knockdown of ADC and ODC1 independently revealed that one of those pathways can compensate for the other for production of polyamines. However, knockdown of both ADC and ODC1 resulted in two-thirds of pregnancies with conceptuses that failed to elongate or produce significant amounts of IFNT. We speculate that the amount of Agm produced by uterine epithelia and rumen bacteria is sufficient for some, but not all conceptuses to survive. Further studies are required to fully assess the phenotype of conceptuses in which translation of mRNAs for both ODC1 and AGMAT is blocked.

## Additional file

Additional file 1: Figure S3. The ovine conceptus transitions from a spherical form on d 12 of pregnancy to a tubular morphology on d 13 and then it elongates progressively in a filamentous morphology to d 16 of gestation. The elongation of the conceptus allows it to establish maximum surface area of attachment to the uterine luminal and superficial glandular epithelia for uptake of nutrients and exchange of gases (oxygen and carbon dioxide) for growth and development, and implantation. (JPEG 122 kb)

## Abbreviations

ADC: Arginine decarboxylase
Agm: Agmatine
AGMAT: Agmatinase
Arg: L-Arginine
IFNT: Interferon tau
MAO: Morpholino
NO: Nitric oxide
OAZ: Antienzyme
ODC1: Ornithine decarboxylase
oTr1: ovine trophectoderm primary cell line
