# Supplementary figures and images for: Functional roles of ornithine decarboxylase and arginine decarboxylase during the peri-implantation period of pregnancy in sheep
Source: J Anim Sci Biotechnol. 2018 Jan 24;9:10. doi: 10.1186/s40104-017-0225-x (PMC5781304; doi:10.1186/s40104-017-0225-x)

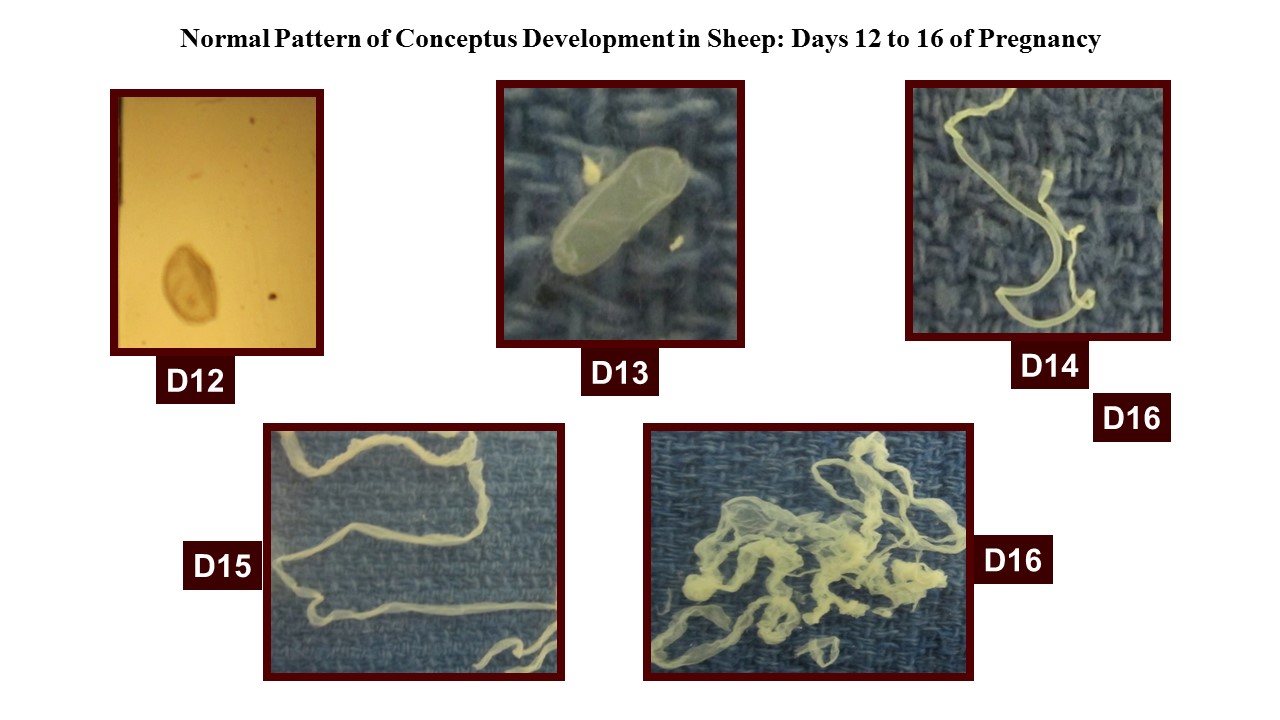

Supplement: Additional file 1: Figure S3. — The ovine conceptus transitions from a spherical form on d 12 of pregnancy to a tubular morphology on d 13 and then it elongates progressively in a filamentous morphology to d 16 of gestation. The elongation of the conceptus allows it to establish maximum surface area of attachment to the uterine luminal and superficial glandular epithelia for uptake of nutrients and exchange of gases (oxygen and carbon dioxide) for growth and development, and implantation. (JPEG 122 kb) [file 40104_2017_225_MOESM1_ESM.jpg]
